# Supplementary material for: Gq neuromodulation of BLA parvalbumin interneurons induces burst firing and mediates fear-associated network and behavioral state transition in mice
Source: Nat Commun. 2022 Mar 11;13:1290. doi: 10.1038/s41467-022-28928-y (PMC8917207; doi:10.1038/s41467-022-28928-y)
Supplement: Supplementary file 1 — Supplementary Figures [file 41467_2022_28928_MOESM1_ESM.pdf]

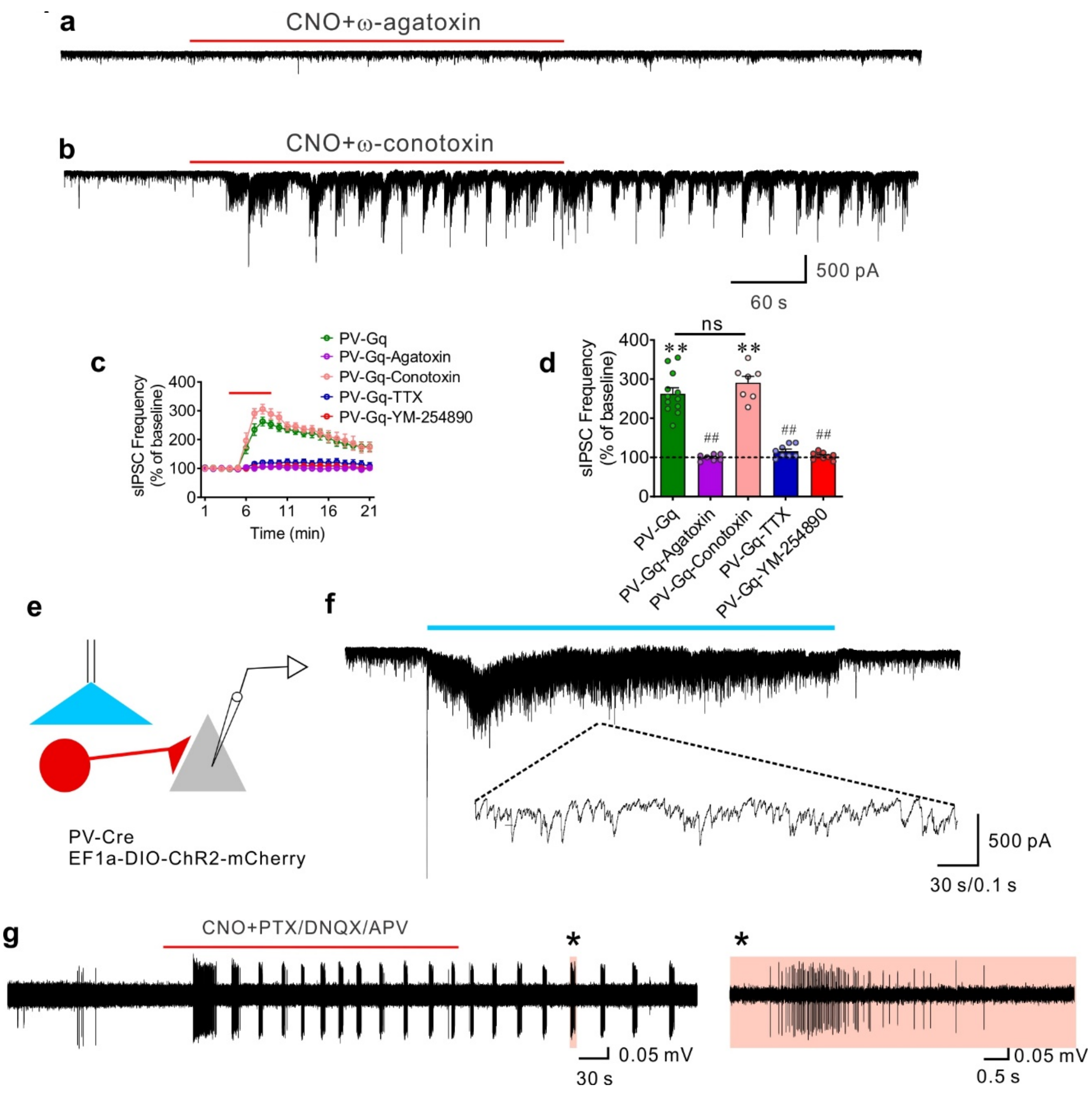

Supplementary Figure 1 (Supporting Figure 1). **a, b** Representative recordings of PV interneuron-mediated repetitive IPSC bursts in a BLA principal neuron, which were blocked by pre-treatment of the slice with the P/Q-type calcium channel blocker ω-agatoxin, but were unaffected by pre-incubation of the slice in the N-type calcium channel blocker ω-conotoxin. **c, d** Time course and mean change (+/- SEM) in sIPSC frequency in response to Gq activation in PV interneurons, which was blocked by ω-agatoxin and insensitive to ω-conotoxin. Pre-treatment of the slice with TTX and YM-254890 abolished the effect of Gq activation in PV neurons on sIPSC frequency. (PV-Gq, n = 12 cells from 5 mice; PV-Gq-Agatoxin, n = 7 cells from 3 mice; PV-Gq-Conotoxin, n = 7 cells from 3 mice; PV-Gq-TTX, n = 8 cells from 4 mice; PV-Gq-YM-254890, n = 9 cells from 4 mice) (Paired *t* test (two-tailed): PV-Gq vs. baseline: $p < 0.0001$, PV-Gq-Conotoxin vs. baseline: $p < 0.0001$, ** $p < 0.01$; One-Way ANOVA, $F(4,38) = 63.35$, $p < 0.0001$; Dunnett's multiple comparisons test, PV-Gq vs. PV-Gq-Agatoxin: $p < 0.0001$, PV-Gq vs. PV-Gq-Conotoxin: $p = 0.27$, PV-Gq vs. PV-Gq-TTX: $p < 0.0001$, PV-Gq vs. PV-Gq-YM-254890: $p < 0.0001$, ## $p < 0.01$, ns not significant). **e** Schematic diagram of recording from BLA principal neurons after transduction and light activation of ChR2 in BLA PV interneurons. **f** Representative recording of the response of a BLA principal neuron to activation of PV interneurons via continuous photostimulation, which generated a tonic increase in IPSCs. Bottom: Expanded trace showing the tonic generation of IPSCs. **g** Representative loose-seal recording of hM3D-Gq expressing BLA PV interneurons showing that the generation of bursting of action potentials in PV cells are not affected by blocking fast excitatory and inhibitory synaptic transmission with GABAA receptor antagonist picrotoxin, AMPA receptor antagonist DNQX and NMDA receptor antagonist APV. The shaded burst marked with an asterisk was expanded to show the accelerating intra-burst spike frequency. Source data are provided as a Source Data file.

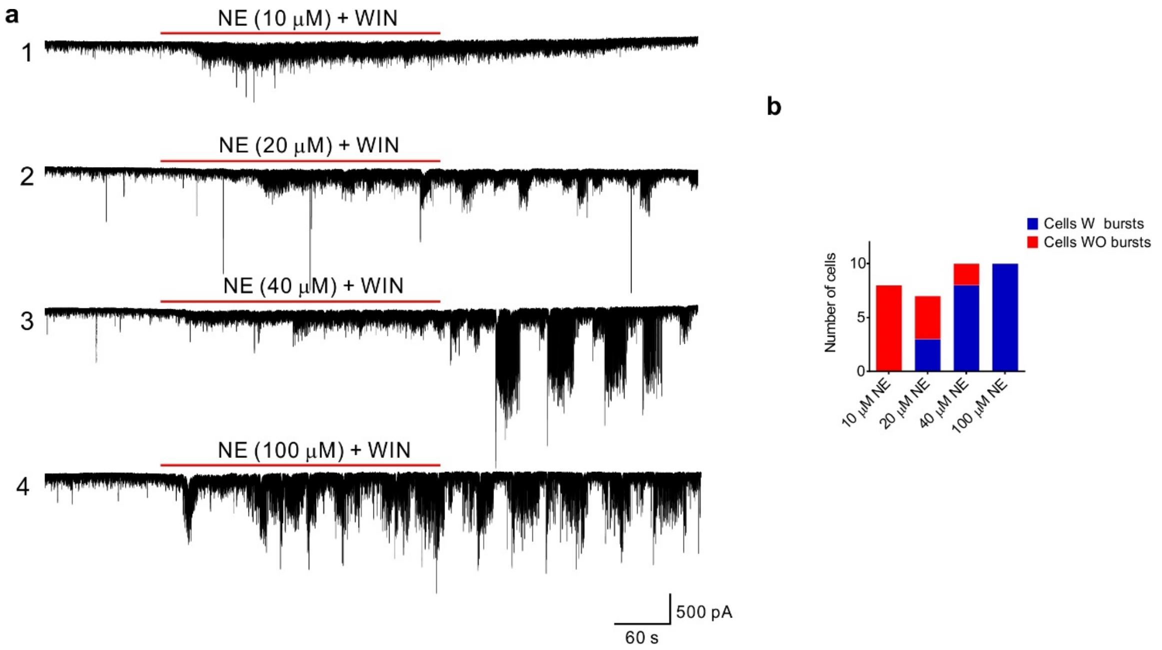

Supplementary Figure 2 (supporting Figure 3). Norepinephrine concentration dependence of sIPSC bursting in BLA principal cells. **a** Representative traces showing the IPSC responses in BLA principal neurons to **1)** 10 µM (8 cells from 3 mice), **2)** 20 µM (7 cells from 3 mice), **3)** 40 µM (10 cells from 3 mice), and **4)** 100 µM NE application (10 cells from 3 mice). The CB1 receptor agonist WIN 55,212-2 (1 µM) was applied to suppress CCK neuron-mediated IPSCs. At concentrations ≥ 20 µM, NE induced repetitive bursts of IPSCs in BLA principal cells. **b** Quantification of the proportion of recorded cells that generated bursts of IPSCs at the different NE concentrations.

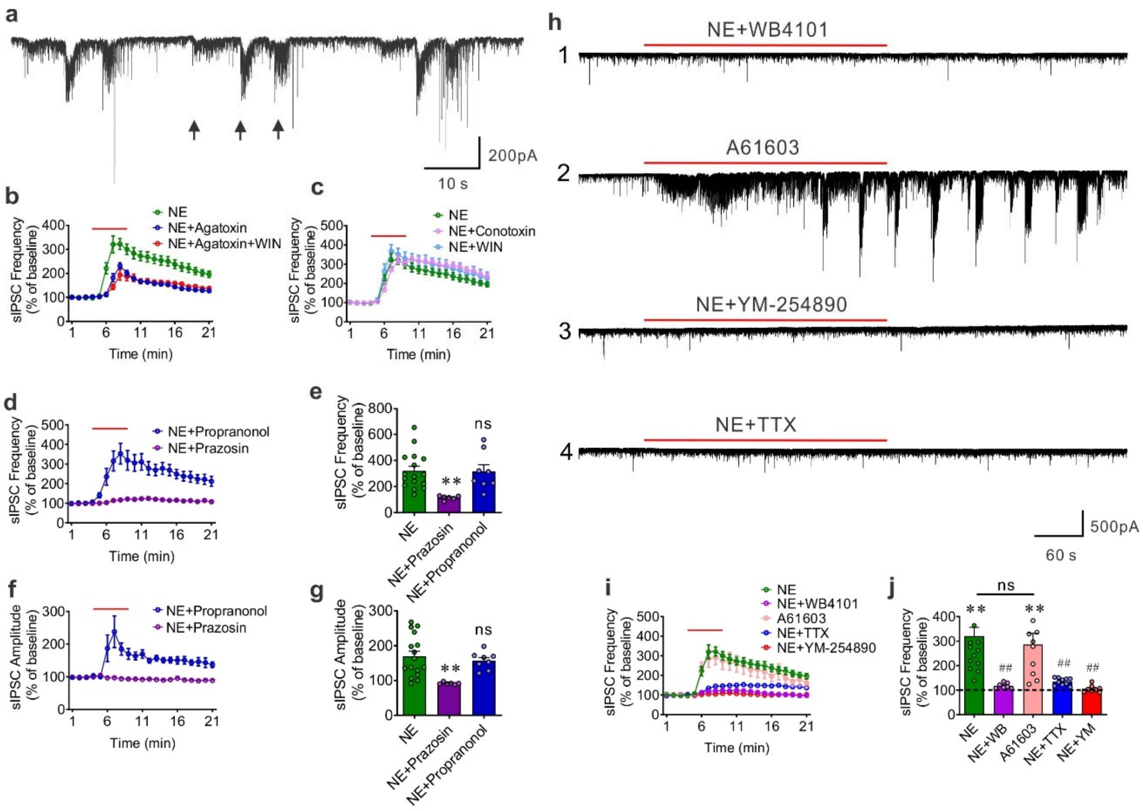

Supplementary Figure 3 (supporting Figure 3). Alpha 1-adrenergic increase in sIPSC frequency and amplitude mediated by presynaptic PV neuron activation. **a** A representative recording of multiple types of NE-induced repetitive IPSC bursts (arrows) recorded in a BLA principal neuron. **b** Time course of the NE-induced change in sIPSC frequency (mean +/- SEM) with and without blockade of P/Q-type calcium channels (ω-agatoxin, n = 10 cells from 4 mice) and activation of CB1 receptors (WIN, n = 8 cells from 3 mice). Blocking P/Q-type calcium channels suppressed the NE-induced increase in IPSC frequency, while the residual frequency response was not further blocked by CB1 activation. **c** Time course of the effect of blocking N-type calcium channels and activating CB1 receptors on sIPSC frequency (mean +/- SEM, n = 12 and 10 cells from 4 and 3 mice, respectively). Neither treatment had any effect on the NE-induced increase in sIPSC frequency, indicating a lack of contribution of CCK interneurons. **d, e** Time course and mean change in sIPSC frequency (Mean +/- SEM) showing that the NE-stimulated IPSCs were blocked by the broad-spectrum α1 adrenoreceptor antagonist prazosin, but were unaffected by the β adrenoceptor antagonist propranolol. (NE: 16 cells from 5 mice; NE + Prazosin: 6 cells from 4 mice; NE + Propranolol: 8 cells from 3 mice) (One-Way ANOVA, F (2, 27) = 6.07, p = 0.0066; Dunnett's multiple comparisons test, NE vs. NE + Prazosin: p = 0.0046, NE vs. NE + Propranolol: p = 0.99, ** p < 0.01, ns not significant). **f, g** Time course and mean change (+/- SEM) in sIPSC amplitude in response to NE in the presence of prazosin and propranolol. Blocking α1 receptors with prazosin abolished the NE effect, but blocking β receptors with propranolol had no effect on the NE facilitation of sIPSC amplitude. (NE: 16 cells from 5 mice; NE + Prazosin: 6 cells from 4 mice; NE + Propranolol: 8 cells from 3 mice) (One-Way ANOVA, F (2, 27) = 6.25, p = 0.0059, Dunnett's multiple comparisons test, NE vs. NE + Prazosin: p = 0.0031, NE vs. NE + Propranolol: p = 0.75, ** p < 0.01, ns not significant). **h** Representative recordings showing that the NE-induced facilitation of sIPSCs depends on activation of presynaptic interneurons through Gq-coupled α1A adrenoreceptors. **(1)** the selective α1A adrenoreceptor antagonist WB4101 blocked the NE-stimulated sIPSCs. **(2)** the α1A adrenoreceptor agonist A61603 induced repetitive bursts of IPSCs following the initial increase of IPSCs, similar to the effect of NE. **(3)** Blocking Gq activity with YM-254890 eliminated the NE-induced increase in sIPSCs. **(4)** Blocking spike activation with TTX prevented the NE facilitation of sIPSCs. **i, j** Time course and mean change (+/- SEM) in sIPSC frequency. The NE-induced increase in sIPSC frequency was blocked by the α1A adrenoceptor antagonist WB4101 and was mimicked by the α1A adrenoceptor agonist A61603. Blocking either spiking activity with TTX or Gq activation with YM-254890 eliminated NE induced increase in sIPSC frequency. (NE: 16 cells from 5 mice; NE + WB4101: 7 cells from 4 mice; A61603: 10 cells from 4 mice; NE + TTX: 10 cells from 5 mice; NE + YM-254890: 8 cells from 3 mice) (paired *t* test (two-sided), NE vs. baseline, p < 0.0001, A61603 vs. baseline: p = 0.0027, ** p < 0.01; one-way ANOVA, F (4, 46) = 10.73, p < 0.0001, Dunnett's multiple comparisons test, NE vs. NE + WB4101: p = 0.0003, NE vs. A61603: p = 0.83, NE vs. NE + TTX: p = 0.0002, NE vs. NE + YM-254890: p < 0.0001, ## p < 0.01, ns not significant). Source data are provided as a Source Data file.

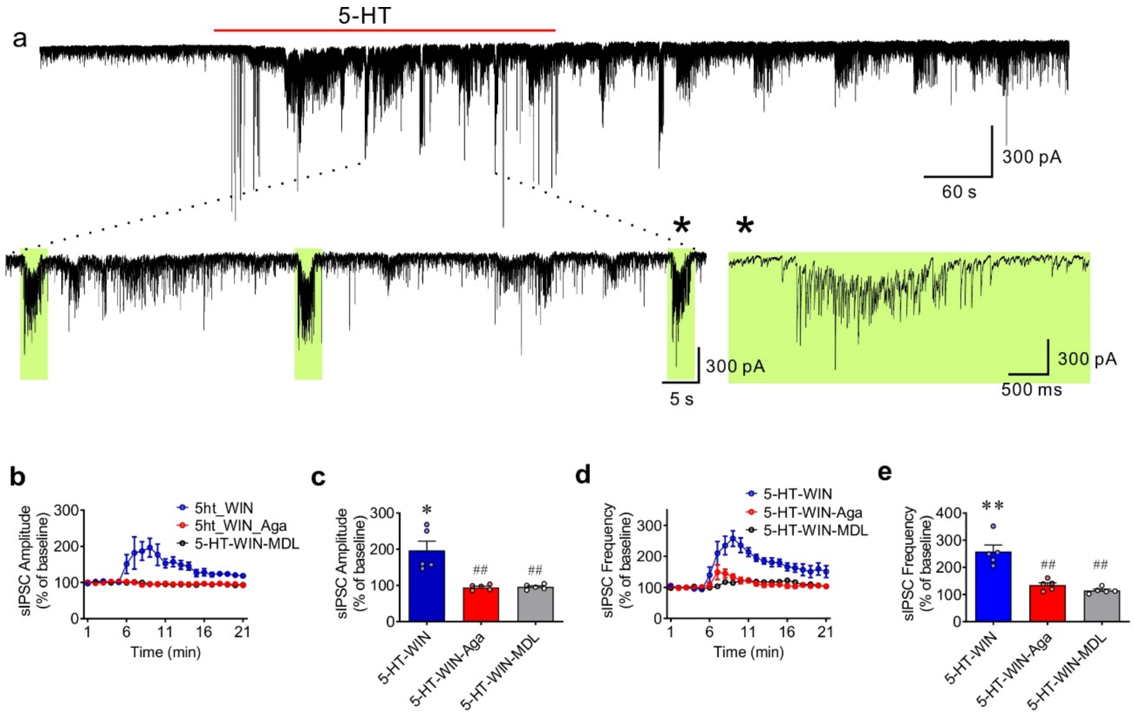

Supplementary Figure 4 (supporting Figure 3). Serotonin 5-HT2A receptor activation of IPSC bursts. **a** A representative recording of serotonin (5-HT, 100 μM)-induced repetitive IPSC bursts in a BLA principal neuron in the presence of glutamate receptor antagonists, DNQX and APV, and the CB1 receptor agonist WIN 55,212-2. Green boxes show three repetitions of the 5-HT-induced bursts. The last repetition was expanded to show the accelerating frequency of intra-burst IPSCs and the negative shift in the baseline due to the progressive summation of high-frequency IPSCs. **b, c** Time course and mean change (+/- SEM) in sIPSC amplitude in response to 5-HT. The 5-HT-induced IPSC bursts were blocked by the P/Q type calcium channel blocker, ω-agatoxin, and the Gq-coupled 5HT2A receptor antagonist, MDL 100907 (1 μM) (5-HT + WIN, n = 5 cells from 3 mice; 5-HT + WIN + agatoxin, n = 5 cells from 3 mice; 5-HT + WIN + MDL 100907, n = 5 cells from 2 mice; paired *t* test (two-sided), 5-HT + WIN vs. baseline, p = 0.023, * p < 0.05; one-way ANOVA, F (2, 12) = 14.77, p = 0.0006, Dunnett's multiple comparisons test, 5-HT + WIN vs. 5-HT + WIN + agatoxin: p = 0.0009, 5-HT + WIN vs. 5-HT + WIN + MDL 100907: p = 0.001, ## p < 0.01). **d, e** Time course and mean change in sIPSC frequency (mean +/- SEM) in 5-HT. Like NE, the 5-HT-induced IPSC bursts were blocked by the P/Q blocker ω-agatoxin and the 5HT2A receptor MDL 100907. (5-HT+WIN, n=5 cells from 3 mice; 5-HT + WIN + agatoxin, n=5 cells from 3 mice; 5-HT + WIN + MDL 100907, n=5 cells from 2 mice; paired *t* test (two-tailed), 5-HT + WIN vs. baseline, p = 0.0034, ** p < 0.01; One-Way ANOVA, F (2, 12) = 24.86, p < 0.0001, Dunnett's multiple comparisons test, 5-HT + WIN vs. 5-HT + WIN + agatoxin: p = 0.0002, 5-HT + WIN vs. 5-HT + WIN + MDL 100907: p < 0.0001, ## p<0.01). Source data are provided as a Source Data file.

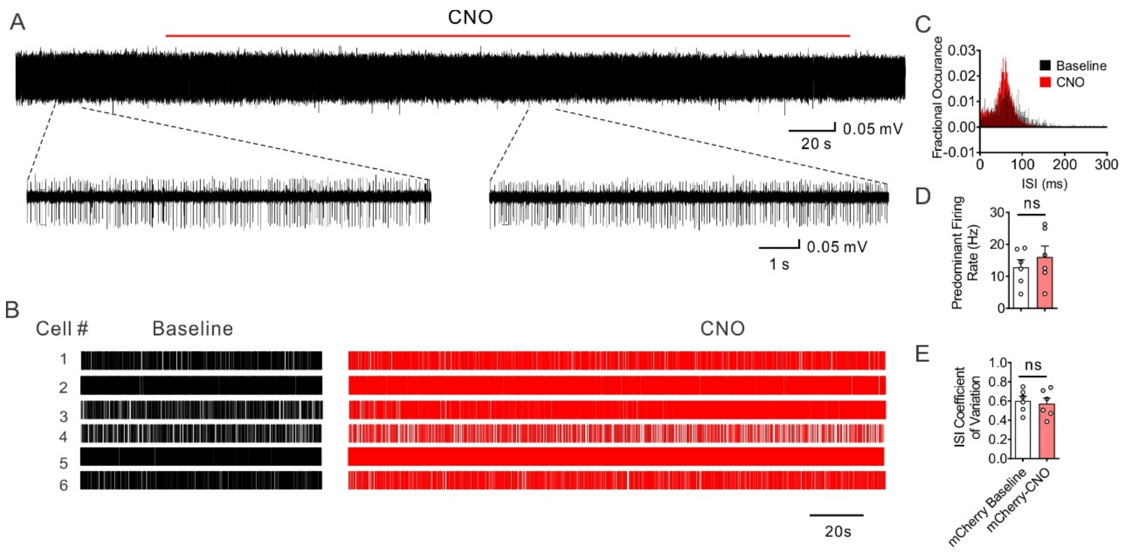

Supplementary Figure 5 (supporting figure 5). CNO application did not alter firing pattern of BLA PV interneurons infected with mCherry alone. **a** A representative recording showing that tonic firing activity of a PV interneuron infected with mCherry alone was not affected by CNO (5µM) application. Below: Traces were expanded to show the tonic firing in the baseline and after CNO application. **b** Raster plots of spiking activity in recordings from 6 control PV interneurons (from 3 mice) expressing mCherry alone that show no transformation of tonic spiking to phasic spiking by CNO. **c** Representative inter-spike interval (ISI) histogram of the cell shown in **a**. **d, e** CNO application in PV neurons expressing mCherry alone did not affect the predominant firing rate **(d)** and ISI coefficient of variation **(e)**. Data are presented as mean +/- SEM; n = 6 cells from 3 mice, paired t tests (two-tailed): predominant firing rate, CNO vs. baseline, p = 0.43; ISI coefficient of variation, CNO vs. baseline, p = 0.18, ns not significant). Source data are provided as a Source Data file.

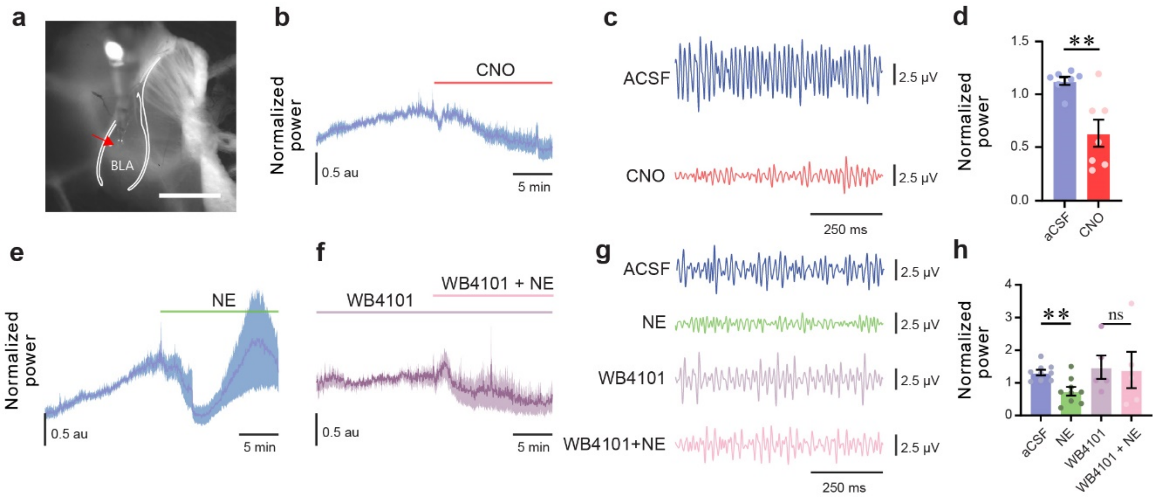

Supplementary Figure 6 (supporting Figure 6). PV Gq signaling reconfigures BLA network activity *ex vivo*. **a** Representative image of the resected BLA slice preparation. Arrow denotes electrode placement. Scale bar, 1 mm. **b** Normalized gamma power (30-80 Hz) area over time before and during CNO activation of virally transduced hM3D-expressing PV neurons in BLA slices from PV-Cre mice. **c** Bandpass filtered (30-80 Hz) raw traces during ACSF (top) or CNO (bottom) application. **d** Mean (+/- SEM) normalized gamma power area across treatments. Paired t test (two-tailed), ACSF vs. CNO (n=7 slices from 4 mice, p = 0.0036). Values from 5 min before and the last 5 min of CNO treatment were used for analysis. ** p < 0.01. **e, f** Normalized gamma power area over time across conditions, **(e)** ACSF, NE, **(f)** WB4101, WB4101 + NE. **g** Bandpass filtered (30-80 Hz) raw traces across treatments (top to bottom: ACSF, NE, WB4101, WB4101+NE). **h** Mean (+/- SEM) normalized power area across treatments. Paired t test (two-tailed), ACSF vs. NE (n = 9 slices from 4 mice, p = 0.0003); WB4101 vs. WB4101+NE (n = 5 slices from 3 mice, p = 0.8706). Values from minutes 3 to 8 were used for analysis (capturing the peak NE-induced gamma suppression). ** p < 0.01, ns not significant, au = arbitrary units.

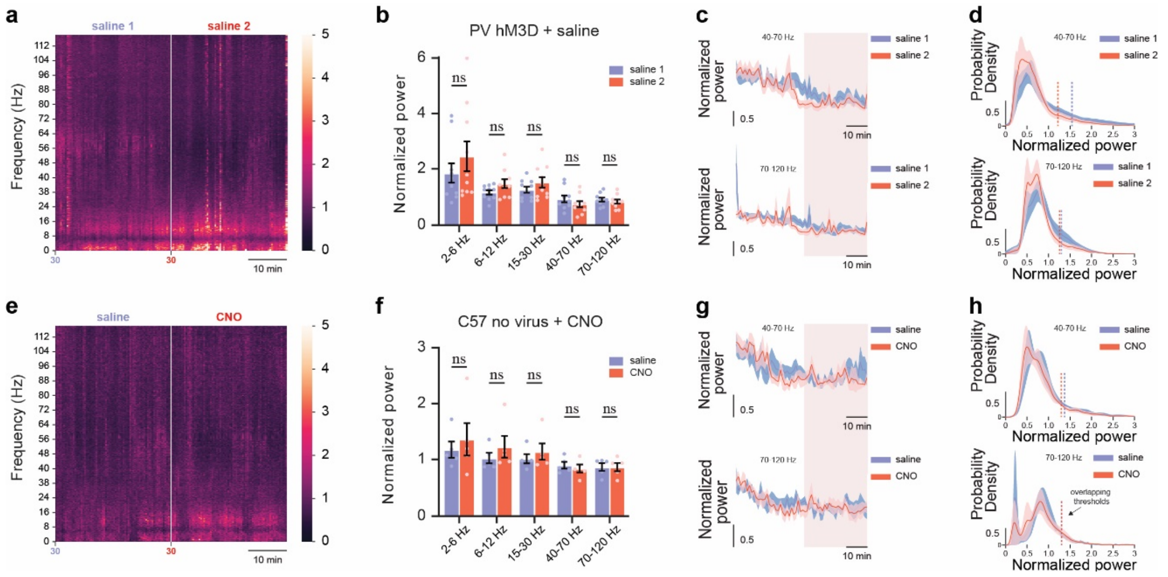

Supplementary Figure 7 (supporting Fig. 6). Neither PV-hM3D + saline nor no virus control + CNO injections reconfigure BLA network activity in vivo. **a** Average spectrogram illustrating normalized power (0-5: low-to-high power) across frequencies over the last 30 minutes of saline 1 and saline 2 treatments in PV hM3D-expressing mice. **b** Mean (+/- SEM) normalized power across treatments (saline 1 vs. saline 2) and frequency bands with individual data points overlaid. Values from last 30 minutes of recording were used for analysis, illustrated by the highlighted sections in **c**. Two-way ANOVA [treatment x frequency], F (1.597, 14.37) = 5.518, p = 0.0215; Sidak's multiple comparisons test: 2-6 Hz, p = 0.2188; 6-12 Hz, p = 0.1347; 15-30 Hz, p = 0.1047; 40-70 Hz, p = 0.5818; 70-120 Hz, p = 0.9222. n = 10 mice. ns not significant. **c** Normalized power across time for 40-70Hz (top) and 70-120 Hz (bottom). Values averaged in 1-min bins. Colored lines = average power over time, colored shaded areas = SEM. Highlighted region indicates last 30 minutes included in analysis. **d** Average probability density plots illustrating the distribution of gamma powers across treatments for 40-70 Hz (top) and 70-120 Hz (bottom). Colored solid lines = average probability, colored shaded areas = SEM. Vertical lines indicate average threshold value for distribution tails, where threshold = 1 standard deviation above the mean. Paired t test (two-tailed), saline 1 vs. saline 2 40-70 Hz distribution tail powers (n = 10 mice, p = 0.3947); saline 1 vs. saline 2 70-120 Hz distribution tail powers (n = 10 mice, p = 0.5975). **e** Average spectrogram illustrating normalized power (0-5: low-to-high power) across frequencies over the last 30 minutes of saline and CNO treatments in C57 no virus control mice. **f** Mean (+/- SEM) normalized power across treatments (saline vs. CNO) and frequency bands with individual data points overlaid. Values from last 30 minutes of recording were used for analysis, illustrated by the highlighted sections in **g**. Two-way ANOVA [treatment x frequency], F (1.133, 4.533) = 1.705, p = 0.2597; Sidak's multiple comparisons test: 2-6 Hz, p = 0.8111; 6-12 Hz, p = 0.5342; 15-30 Hz, p = 0.5584; 40-70 Hz, p = 0.6465; 70-120 Hz, p > 0.9999. n = 5 mice. ns not significant. **g** Normalized power across time for 40-70 Hz (top) and 70-120 Hz (bottom). Values averaged in 1-min bins. Colored lines = average power over time, colored shaded areas = SEM. Highlighted region indicates last 30 minutes included in analysis. **h** Average probability density plots illustrating the distribution of gamma powers across treatments for 40-70 Hz (top) and 70-120 Hz (bottom). Colored solid lines = average probability, colored shaded areas = SEM. Vertical lines indicate average threshold value for distribution tails, where threshold = 1 standard deviation above the mean. Paired t test (two-tailed), saline vs. CNO 40-70 Hz distribution tail powers (n = 5 mice, p = 0.4751); saline vs. CNO 70-120 Hz distribution tail powers (n = 5 mice, p = 0.6763). Source data are provided as a Source Data file.

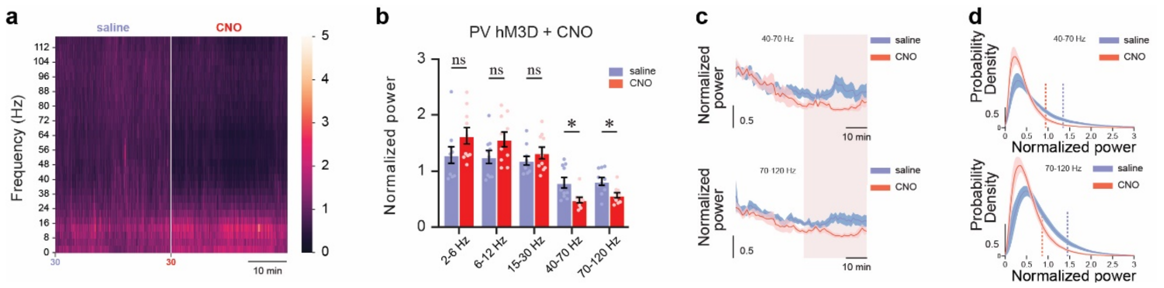

Supplementary Figure 8 (supporting Figure 6). 0.25 second (10 cycle) FFT analysis window produces similar results to the 5 second window analysis. **a** Spectrogram illustrating normalized power (0-5: low-to-high power) across frequencies over the last 30 minutes of saline and CNO treatments in PV hM3D-expressing mice. Note that less than 10 cycles will be captured for frequencies below 40 Hz with this 0.25 second window size. **b** Mean (+/- SEM) normalized power across treatments (saline vs. CNO) and frequency bands. Values from last 30 minutes of recording were used for analysis, illustrated by the highlighted segments in **c**. Two-way ANOVA [treatment x frequency], F (1.058, 9.523) = 6.970, p = 0.0246; Sidak's multiple comparisons test: 2-6 Hz, p = 0.2857; 6-12 Hz, p = 0.3196; 15-30 Hz, p = 0.6891; 40-70 Hz, p = 0.0326; 70-120 Hz, p = 0.0135. n = 10 mice. * p < 0.05. ns not significant. **c** Normalized power across time for 40-70 Hz (top) and 70-120 Hz (bottom). Values averaged in 1-min bins. Colored lines = average power over time, colored shaded areas = SEM. Highlighted region indicates last 30 minutes included in analysis. **d** Average probability density plots illustrating the distribution of gamma powers across treatments for 40-70 Hz (top) and 70-120 Hz (bottom). Colored solid lines = average probability, colored shaded areas = SEM. Vertical lines indicate average threshold value for distribution tails, where threshold = 1 standard deviation above the mean. Paired t test (two-sided), saline vs. CNO 40-70 Hz distribution tail powers (n = 10 mice, p = 0.0042); saline vs. CNO 70-120 Hz distribution tail powers (n = 10 mice, p = 0.0009). Source data are provided as a Source Data file.

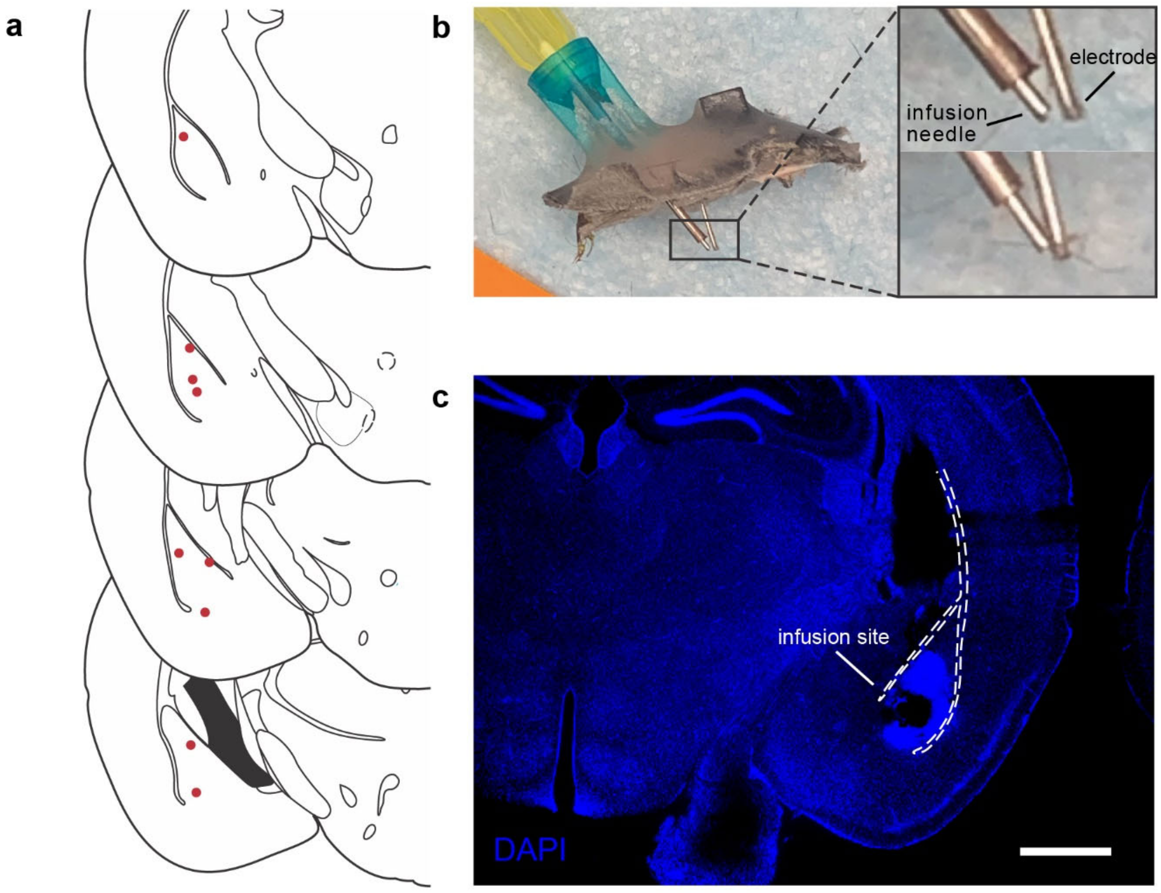

Supplementary Figure 9 (supporting Fig. 7). Intra-BLA infusion cannula placement. **a** BLA cannula placement across mice. **b** Extracted representative implant showing electrode placement relative to microinfusion cannula. Right: higher magnification images of two implants illustrating the variable distance between the microinfusion cannula and LFP recording electrode. **c** Coronal DAPI-stained BLA section demonstrating BLA cannula placement accuracy. The lesioned area indicates the infusion site within the BLA, which is demarcated by the dashed lines. Scale bar, 1 mm.

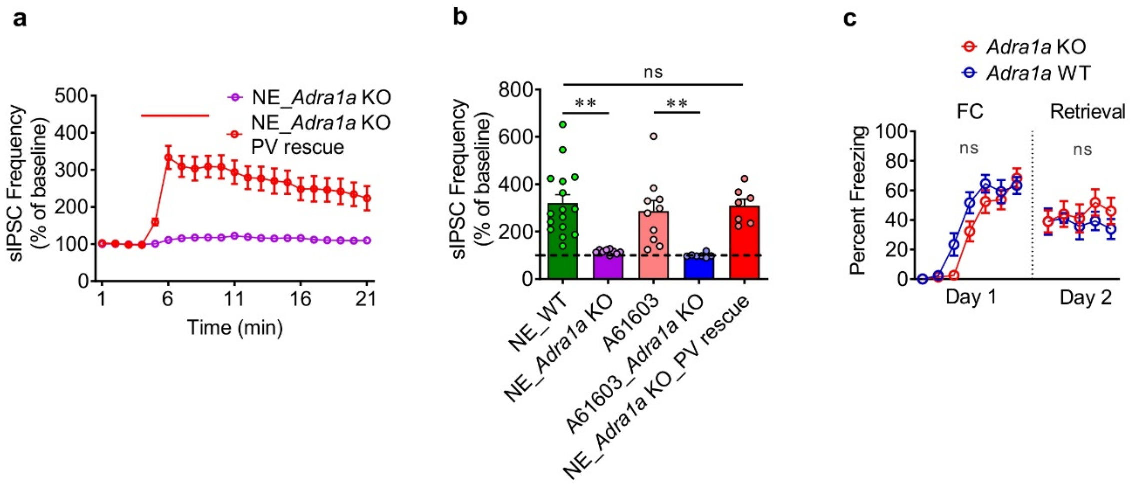

Supplementary Figure 10 (supporting Figure 8). Effect of *Adra1a* knockout and rescue on NE-induced increase in sIPSC frequency. **a** Time course of NE effect of sIPSC frequency (mean +/- SEM) in *Adra1a* KO mice with or without re-expression of α1A adrenoreceptors in BLA PV interneurons (NE_*Adra1a* KO: 9 cells from 4 mice; NE_*Adra1a* KO PV rescue: 7 cells from 3 mice). **b** Mean change (+/- SEM) in sIPSC frequency showing that rescue of α1A noradrenergic signaling in BLA PV interneurons restored the NE facilitation of sIPSC frequency that was largely lost in BLA principal neurons in slices from *Adra1a* KO mice (NE_WT: 16 cells from 5 mice; NE_*Adra1a* KO: 9 cells from 4 mice; A61603_WT: 10 cells from 4 mice; A 61603_*Adra1a* KO: 7 cells from 3 mice; NE_*Adra1a* KO_PV rescue: 7 cells from 3 mice) (unpaired *t* test (two-tailed), A61603_WT vs. A61603_*Adra1a* KO: p=0.0037; One-Way ANOVA, F (2, 29) = 11.43, p = 0.0002, Dunnett's multiple comparisons test, NE_WT vs. NE_*Adra1a* KO: p = 0.0002, NE_WT vs. NE_*Adra1a* KO_PV rescue: p = 0.96, ** p < 0.01, ns not significant). **c** Global knockout of α1A does not have significant effects on fear memory acquisition (n = 12 *Adra1a* KO mice and n = 10 littermate WT mice, Mean +/- SEM, Two-Way repeated measures ANOVA, F (1, 20) = 2.49, p = 0.13 compared to WT littermate controls), and on fear memory retrieval (mean +/- SEM, Two-Way repeated measures ANOVA, F (1,20) = 0.60, p = 0.45), ns not significant. Source data are provided as a Source Data file.
